# They are my worries, so it’s me the doctor should listen to—adolescent males’ experiences of consultations with general practitioners

**DOI:** 10.1186/s12875-024-02431-3

**Published:** 2024-05-17

**Authors:** Johanna Haraldsson, Linus Johnsson, Ylva Tindberg, Per Kristiansson, Lena Nordgren

**Affiliations:** 1 Department of Public Health and Caring Sciences/Family Medicine and Preventive Medicine, Uppsala University, Box 564, SE-751 22 Uppsala, Sweden; 2 Centre for Clinical Research Sörmland / Uppsala University, Mälarsjukhuset, SE-631 88 Eskilstuna, Sweden; 3 Centre for Research Ethics & Bioethics, Uppsala University, Box 564, Uppsala, SE-751 22 Sweden; 4 Department of Women’s and Children’s Health, Uppsala University, Akademiska sjukhuset, SE-751 85 Uppsala, Sweden; 5 Department of Public Health and Caring Sciences/Caring Sciences, Uppsala University, Box 564, SE-751 22 Uppsala, Sweden

**Keywords:** General practitioners, Family practice, Physician-patient relations, Adolescent medicine, Qualitative research, Adolescents, Sweden

## Abstract

**Background:**

Many adolescent males visit a general practitioner regularly, yet many report unmet health needs and negative experiences. This indicates a gap between provided healthcare and the needs of adolescent males. In order to improve adolescent males’ possibilities to discuss their health concerns with general practitioners, the study’s aim was to explore and describe how adolescent males understand and assign meaning to their experiences of consultations with general practitioners.

**Methods:**

This qualitative study was conducted at two healthcare centres in mid-Sweden in 2022. Nine males 15 to 19 years old described their experiences in semi-structured interviews immediately after consulting a general practitioner, regardless of reason for the consultation and whether or not accompanied by a parent. The analysis was guided by thematic analysis according to Braun & Clarke and reflective lifeworld theory’s concepts of openness and sensitivity.

**Results:**

One overarching theme, *To be listened to*, and three themes were developed: *To handle insecurity and uneasiness, To be understood and cared for*, and *To get parental support on his terms*. In a good appointment, the general practitioner cares about him, listens attentively, and takes him seriously. More importantly, the general practitioner’s understanding permeates the consultation, so that all aspects of it is adapted to him. The adolescent males doubted their ability to express themselves and to understand what would happen in the consultation, and therefore feared being dismissed without receiving any help. Such difficulties may be due to unfinished neurocognitive development and inexperience. They struggled with embarrassment, partly due to notions of masculinity, and strived to balance their needs of parental support, privacy, and being the one that the doctor listens to.

**Conclusions:**

We argue that adolescent males are particularly vulnerable due to on-going neurocognitive and emotional development, inexperience, and notions of masculinity. However, good experiences can be generated through rather simple means. Adolescent males need individual adaptations demonstrating that they are cared for, understood and taken seriously. Furthermore, they need an unhurried pace to facilitate understanding, verbal affirmations to mitigate embarrassment, and help in navigating parental involvement.

**Supplementary Information:**

The online version contains supplementary material available at 10.1186/s12875-024-02431-3.

## Background

Many 15- to 19-year-old males visit a general practitioner (GP) every year [1–4], yet report needing more guidance, particularly in the areas of lifestyle and mental health [1, 5, 6]. For several reasons, such concerns risk not being disclosed to the GP. Adolescents may doubt whether lifestyle and mental health concerns are appropriate to discuss with the GP [7–9], conceiving of the GP as a resource for physical health only [3, 7–11]. They also report difficulties talking about sensitive issues with GPs [7, 9, 10], struggling to find the right words or to explicitly express their concerns [7, 10]. Altogether, this indicates missed opportunities for discussing the adolescents’ own health concerns in GP consultations.

GPs describe consultations with adolescents as professionally challenging, perceiving them as unwilling to disclose the full extent of their concerns [12, 13]. GPs can ameliorate difficulties talking about sensitive issues by offering confidentiality, i.e. private time with the GP in the absence of parents and an explanation of what professional secrecy entails [2, 6, 14, 15]. Besides confidentiality, many adolescents want honest and respectful dialogues with an empathic, accepting, non-judgmental GP [3, 7, 10, 15–17], and opportunities to ask questions [15]. Yet, adolescents describe in some, but not all, studies experiences of meeting unengaged and dismissive GPs who made them feel disbelieved and not listened to [7, 8, 10, 18]. Such findings clash both with their right to be listened to [19, 20] and with the emphasis that GPs themselves place on the importance of responding to the patients’ needs [21], indicating that GPs sometimes fail in their intended efforts.

Compared with their female peers, the situation is more problematic for adolescent males for several reasons. First, adolescent males run higher health risks; they have twice the mortality rate, mostly due to lifestyle and mental health adversities [22, 23], yet use less healthcare [24]. Second, adolescent males seem to struggle more with disclosing sensitive information, such as mental health, than their female peers [25–28]. Third, there are indications of physicians communicating less well with them [29]. However, how adolescent males experience GP consultations is still unclear. Compared with females, they particpate to a lesser extent in qualitative studies [30], and few studies focus on the perspectives of adolescent males only [27].

Research on adolescents’ relationships with GPs is scarce [9]. To the best of our knowledge, the reason for the discrepancy between adolescent males’ experiences and GPs’ intents is unknown. To improve their encounters with GPs, a deeper understanding of adolescent males’ experiences is required [7]. Such an understanding can be achieved through an inductive, lifeworld-based approach in which a deliberately wide aim allows openness in the face of unexpected findings [31]. The aim of the study was therefore to explore and describe how adolescent males understand and assign meaning to their experiences of GP consultations.

## Methods

A qualitative research approach was chosen to explore and describe how experiences are understood and assigned meaning to.

### Design and setting

The present study is the first part of a larger project focusing on adolescent males’ GP consultations in mid-Sweden. The consultations were video-recorded and followed by interviews with the adolescent males and the GPs, respectively. Notably, all participating GPs were not fully trained GPs. Some of them were residents in general practice, or junior doctors, but they all worked as GPs, and were thus probably perceived as GPs by the adolescent males. This paper is based only on the interviews with the adolescent males.

### Population and participants

All males born in 2002 to 2006 who, for any reason, visited any of the GPs included in the project were invited to participate in the study. The study aimed to explore experiences of adolescent males 15 to 19 years old, but in order to keep the inclusion criteria identical throughout the data gathering period, year of birth was chosen instead of actual age. Exclusion criteria were inability to communicate in Swedish (i.e., need of an interpreter) or severe acute illness. Three adolescent males were excluded due to insufficient language skills, and none due to severe illness. No other inclusion or exclusion criteria were used.

Data were collected from 4 March to 20 May, 2022, in two public healthcare centres in Region Sörmland, Sweden. Healthcare centre A is situated in a small town which, compared with Sweden as a whole, has slightly higher levels of income, employment rate, and fraction of adolescents fulfilling requirements for upper secondary school when leaving compulsory school. Healthcare centre B is located in a medium-sized town with a large population born outside Europe, a high rate of unemployment, and a lower proportion of adolescents accomplishing compulsory school [32].

### Ethical considerations

Before participation, all informants received oral and written information about the aim and procedure of the study, and completed a written informed consent. According to Swedish law, no parental consent is needed for study participation for adolescents above the age of 15 [33]. The Swedish Ethical Review Authority approved the study design (Dnr 2022-00075-01).

### Data gathering

Ordinary healthcare staff contacted eligible males a few days before their scheduled appointments. Those who were interested in the study were thereafter invited by phone by JH. If unreachable by phone, which could be the case in urgent consultations, they were invited on arrival to the healthcare centre. The number of participants was not decided beforehand. Instead, the richness and variation of data relevant to the research question were evaluated at the end of the predefined data gathering period. The last two interviews had added only a limited amount of new aspects, and sufficient information power was deemed to have been attained at that point [34, 35]. Thus, no more data gathering periods were initiated.

Of fifteen invited adolescent males, nine wanted to participate (mean age 17.3 years; Table 1). The nine consultations were video-recorded (for another study). The adolescent male and the GP mutually decided whether the researcher (JH) was allowed to stay in the consultation room. In all consultations except one, JH started the video recording and then left.

**Table 1:** Presentation of the participating adolescent males

• *Tomas*^a^, a Swedish-born 17-year-old visited GP1 (a young female at healthcare centre A) because of an ingrowing toe nail. He was accompanied by his girlfriend^b^.
• *Perzy*^a^, a Swedish-born 15-year-old visited GP1 alone because of tinnitus.
• *Nasir*^a^, a Swedish-born 17-year-old visited GP1 alone because of hair loss. Both of his parents were born outside Europe.
• *Ali*^a^, a foreign-born 17-year-old visited GP2 alone (a middle-aged male at healthcare centre A) because of snoring. He was born outside Europe, as were both of his parents.
• *Anton*^a^, a Swedish-born 17-year-old visited GP2 because of a sore throat. He was accompanied by his mother^b^.
• *Axel*^a^, a Swedish-born 18-year-old visited GP3 (a young male at healthcare centre B) because of tiredness. He was accompanied by his father^b^, who was asked by the GP to step outside during the physical examination.
• *Erik*^a^, a Swedish-born 16-year-old visited GP4 (a young male at healthcare centre B) because of impaired hearing. He was accompanied by his father^b^.
• *Majed*^a^,a foreign-born 20-year-old visited GP5 (a young female at healthcare centre B) alone because of hair loss. He was born outside Europe, as were both of his parents. (Majed was born early in 2002, and was included as a result of using year of birth instead of actual age as inclusion criterion).
• *Carl*^a^, a Swedish-born 19-year-old visited GP6 (a young female at healthcare centre B) alone because of a cough.

^a^The pseudonyms were chosen by the adolescent males themselves, but some had to be adjusted to better protect their identity

^b^Relatives are noted as accompanying if attending the consultation. Adolescent males that came unaccompanied or had relatives that never entered the consultation room are described as visiting the GP alone

Immediately after the consultation, individual, semi-structured interviews were conducted with the adolescent males about their experiences of the consultation. The interviewee and JH were seated opposite each other in a vacant consultation room at the healthcare centre. Accompanying parents or partners waited outside. However, due to a sore throat, one participant was interviewed by phone two weeks after the consultation. The nine interviews were digitally audio-recorded and lasted between nine and thirty-two minutes (median 20 minutes). All interviews were conducted in Swedish and started with the same question: *You have just met Dr X. How was it?* An interview guide developed for this study was used, covering the following topics, although the order and the wording of the open-ended questions varied: the consultation, the doctor, communication, presence of relatives, and possible improvements (Supplement 1). The answers were followed up with clarifying questions like: *Why is that important to you?* and *How do you mean?* The interview guide was not pilot tested, but the semi-structured format allowed JH to continually improve and individualize the discussions. The adolescent males were asked to compare the doctor with previously encountered ones, which yielded several descriptions of prior unsatisfying consultations in primary care and elsewhere (compiled in Table 2). This turned out to be a fruitful path towards understanding the “good” in a good appointment, because by contrasting the bad experiences with the good ones, the adolescent males could more deeply describe what was important to them. After the interview, a short note was written about it. The adolescent males were rewarded with two cinema tickets each.

**Table 2:** Contrasting with unsatisfying appointments

Although the adolescent males reported being pleased with the studied appointments, they also described previously experienced unsatisfying appointments in primary care and elsewhere. These appointments are out of the scope of the present study due to their variation in location (primary care or hospitals) and time (during childhood or adolescence). As these experiences enriched the descriptions and might have affected the adolescent males’ perceptions of the studied consultation, they are here presented in a condensed and slightly interpreted form, as non-thematic contextualising information [36].
An unsatisfying appointment consists in being ignored, dismissed and belittled. It is a neglectful and unprofessional appointment, in which he wastes his own and the doctor’s time. In an unsatisfying appointment, his lifeworld and his experiences of the concern are deemed irrelevant. He feels stupid, falls silent, and loses hope of getting any help. Advice and treatment demonstrate that the doctor has not understood the problem. An unsatisfying appointment means being abandoned without the help needed, and he feels sad and angry.
*They* [the doctors] *all do the absolute minimum … so they can hurry you back out there again … so they can go back to drinking coffee or whatever. It all felt so … so, kind of like “why did I even bother coming here, they don’t give a shit”, ‘cos when I entered my appointment he literally said that it was pretty much an unnecessary visit. (Erik, 16)*
*She* [the doctor] *spoke so fast that I could barely hear what she was saying. And then she ran around all over the place fetching stuff. I couldn’t quite keep up. (Tomas, 17)*

### Analysis

The data were analysed inductively with reflexive thematic analysis according to Braun and Clarke [37–39]. Thematic analysis is a flexible way to seek patterns across a set of data, and can generate unanticipated insights and rich descriptions [37, 39]. It can be used in both inductive and deductive approaches and together with various theoretical perspectives [37, 39], such as lifeworld theory [40]. In the present study, thematic analysis was chosen because of the authors’ intent to inductively develop and describe patterns in the adolescent males’ lived experiences. According to our ontological and epistemological framework, the lifeworld theory, we all live in a shared world, but each of us experiences it differently [31]. Thus, all of us have our own unique lifeworld. Exploring this world of lived experiences and meanings requires openness and sensitivity, so that we can approach the phenomenon with curiosity striving to see all its multiplicity including unanticipated meanings. Understanding is bridled, i.e. presupposed assumptions are recognized and questioned, but kept aside so that they do not lead understanding astray [31]. A bridled attitude means that the whole research process is scrutinized and the development of understanding is reflected upon, in order to be true to the phenomenon and avoid hasty conclusions [31]. Thus, in thematic analysis of lived experiences a reflective and questioning approach is used to illuminate patterns of shared meaning, as well as guide the whole research process [40].

In thematic analysis, the goal is to develop distinctive, clearly separated themes that simultaneously share a central unifying concept, thus telling a coherent overall story [37–39]. The analysis was guided by the six phase process described by Braun & Clarke [37, 39] and by the ambition towards openness prescribed by reflexive lifeworld research by Dahlberg [31, 40]. JH transcribed the interviews verbatim and checked the transcripts against the original audio files. JH and LN read the text thoroughly until the content was perceived as familiar (phase 1). JH and LN condensed meaningful data extracts and gave each of them one or more initial codes, depending on the richness of content (phase 2). Every time a new code was created, already coded data were revisited in search of the new code. In a process that moved between description and interpretation, JH, LN, and LJ grouped the codes, searched for patterns, generated candidate themes, and created a thematic map (phase 3). JH wrote down the core concepts in the data to find out the scope of and the limits between the candidate themes. JH, LN, and LJ checked the themes against codes, condensed data extracts, and the whole data set, and altered them accordingly (phase 4). Thereafter, the themes were named, further revised and discussed thoroughly among JH, LN and LJ (phase 5). JH reviewed notes from data gathering and analysis and tied up loose ends. Finally, the themes were drafted in a manuscript, illustrated with data extracts, and refined in discussions among all authors (phase 6).

## Results

**Fig. 1:**
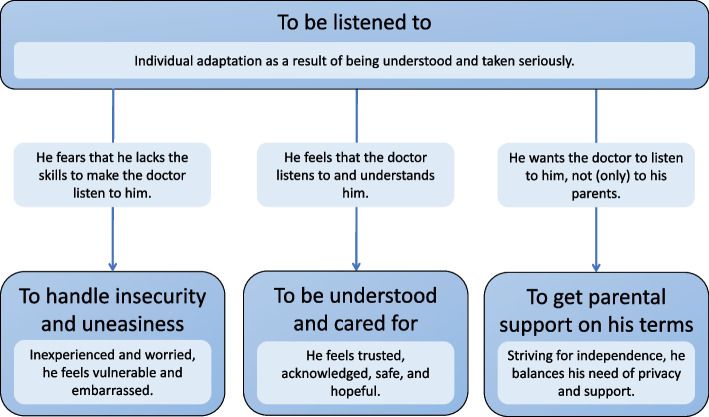
Thematic map demonstrating the four themes. The over-arching theme, *To be listened to,* tells the overall story, while the three themes *To handle insecurity and uneasiness*, *To be understood and cared for*, and *To get parental support on his terms* can be seen as three different chapters in that story. The central aspects of each theme is briefly described. Each arrow is labelled to indicate the relationship between the overarching theme and the subordinate themes.

Overall, the adolescent males reported that they were happy with their appointments. All felt listened to and described the doctor in positive phrasing such as *good*, *really kind*, and *professional*. The overarching theme telling the overall story was called *To be listened to*. Furthermore, three themes were developed; *To handle insecurity and uneasiness, To be understood and cared for*, and *To get parental support on his terms* (Fig. 1).

### To be listened to

To be listened to means to be understood and taken seriously. It means meeting an interested doctor who listens carefully and attentively, sincerely trying to understand how he experiences his troubles. When he is being listened to, the consultation and advice are adapted to his needs and to him as an individual. The medical world is connected to his lifeworld and he dares to hope for a helpful solution. Due to his young age and inexperience, being listened to cannot be taken for granted. As a result, he is sometimes in need of support. However, bringing a supporting parent also entails the risk of the doctor listening more to his parent than to himself.

### To handle insecurity and uneasiness

Insecurity means that he, unfamiliar with being a patient, is unsure of how to behave and what to expect of a doctor’s appointment. It is a novel situation, in which he might worry about his concerns and the medical procedures he envisions. Insecurity also means to doubt his communication skills, not trusting his own ability to explain well enough to be understood correctly or to understand what the doctor means.

When she [the GP] asks a question … and I don’t know how to answer, and … well I start to sort of giving answers at random, making guesses… (Perzy, 15)

Uneasiness is about potentially embarrassing situations in which he exposes his vulnerability, such as when taking off his clothes or when he does not understand the doctor. It means being in a strange situation where he is supposed to expose himself and reveal his worries. He struggles with being vulnerable while upholding his role as a man, as he has been taught that men never reveal themselves as frightened, sensitive, or in pain.

They [men] don’t want to talk about sensitive stuff like that … and, you know, guys will usually shy away from being too sensitive … from an early age they’ve been told, “Don’t be sensitive, don’t cry, don’t do things like that, don’t show any feelings”. (Majed, 20)

Insecure in meeting the expectations in how to communicate and behave, he worries about not being listened to nor taken seriously, but instead being neglected or humiliated. He would then have wasted his and the doctor’s time and revealed himself as vulnerable in vain. Thus, he fears to be dismissed without help due to his youth and lack of experience.

Insecurity and uneasiness can be handled in various ways, for instance by comforting thoughts, taking action during the consultation, or preparations. He might trust the doctor to know what to do, or he might tell the doctor about his fears and wished-for adjustments. He might prepare the appointment beforehand at home with his parents. He might also bring a parent or a partner along for support, who can act on his behalf, or just be there, silently providing safety.

I thought it was nice to have him [dad] tag along … because he … we do talk a lot beforehand about, like, all this … but then, when I’m supposed to come and tell, I forget … I get so nervous … eh, yeah, so it’s comforting to have him there. (Axel, 18)

### To be understood and cared for

To be understood and cared for is about feeling acknowledged, safe, and hopeful. It means that his worries are correctly interpreted and that he feels trusted and taken seriously. The doctor makes clear that he or she sincerely want to understand him and his concerns. The doctor devotes sufficient time, asks about all conceivable details, and checks his or her comprehension by inviting him to correct misunderstandings.

She [the doctor] even let me correct her, and it … it’s not like she just says things … no, while she spoke, she paused and looked at me, to make sure that it … that she was on the right track … It felt like she wanted me to correct her if she had gotten it wrong. (Tomas, 17)

The doctor demonstrates his or her understanding of his troubles and his lifeworld by adapting the appointment, advice, and treatment to his needs. The doctor adjusts his or her language, pace*,* and actions, so that he has time to grasp what is being said and takes place, and to ask if he does not. He understands what is wrong and how he can handle his problem. He gets advice that he deems helpful and that fits into his everyday life with school and friends, or if it does not, a possibility to influence the doctor’s decision.

If I, like … tell the honest truth about how things are … and then he [the doctor] adapts to what I have said … it pleases me, that he does his best to like, listen to what I’ve told him and forms his opinion based on that … he checks out how things are and stuff like that, and then tells me how it is … (Erik, 16)

To be understood and cared for means that the doctor cares about him and that he feels safe. The doctor is interested, engaged, and makes an effort for his benefit. The doctor appears knowledgeable and skilled, and makes clear that he or she truly want to help him, for instance by offering a follow-up to ensure that he gets better. He is comfortable talking to the doctor. The doctor is kind, nice, treats him respectfully, for example by listening without interrupting. He is at ease, dares to open up, and to talk about his body. He can venture other complaints, at least when important enough and within the doctor’s field of knowledge. When feeling cared about and safe, he can trust the doctor.

It was just like chatting to anyone … you know … you listen to each other. You don’t interrupt each other. That’s mostly it, the way I think about it … listen to what the other one is saying and don’t interrupt. (Tomas, 17)

To be understood and cared for means feeling hope. When the doctor has listened to him and comprehended not only his medical problem but also how he experiences his situation, he dares to trust the doctor to find a helpful solution. He draws hope from alternative treatment options being available or, when further examinations appear to be necessary, the fact that the doctor has recognized that something has to be done. When leaving the appointment, he feels relieved, optimistic, less worried, and hopeful of an end to his troubles.

She [the doctor] made me feel more comfortable when she told me not to worry about these kinds of things, that there’s always a reason why they happen and that you can always find the answer. So, she really took away my worries, if you can put it that way. (Nasir, 17)

### To get parental support on his terms

To get parental support on his terms means balancing between independence and need of parental support. On the one hand he needs privacy and to be the one the doctor listens to, and on the other hand he needs his parents’ help in practical matters, or just for them to be there for safety. How parental presence is perceived varies within and between the studied appointments, and is described as irrelevant, insignificant, supportive or inhibiting. This suggests that, to be beneficial, parental presence must be on his terms.

His independence is manifested in terms of no longer fully trusting his parents’ medical advice. Instead, he wants to discuss his concerns with a doctor, and therefore gets himself, with or without the help of his parents, a doctor’s appointment. To visit the doctor on his own comes naturally, and he might perceive parental presence as irrelevant.

Parental presence can also be described as insignificant. It is neither needed, nor unwelcome, but merely taken for granted and a sign of that his parents care about his health. The parents do not affect the appointment, or whether he feels free to ask questions. They just sit there, listening.

It wouldn’t make any difference, because dad would just sit there and listen. (Ali, 17)

Supportive parental presence means that the parents are welcome to accompany him, because they provide appreciated support and safety, but also practical assistance, for instance by answering questions about his early childhood.

He [the doctor] was asking the questions, I answered, but then mum … elaborated … (Anton, 17)

Parental presence can be perceived as inhibiting when he needs privacy. He might be embarrassed, or just want to keep his parents in the dark about private issues, such as sexual activities or use of tobacco or alcohol. Nor does he want to discuss issues involving his parents in their presence. He might handle the situation by lying or phrasing himself carefully. He might also, as a way of taking responsibility for his own health, discuss the embarrassing issues in a straight and honest manner, trusting his parents not to lecture him about them. Inhibiting parental presence can also mean that the parents take over the discussion so that their view of the problem comes to overshadow his own. When he is alone, the doctor listens more attentively to him and how he is affected by the problem. Thus he emphasizes that, because it is his worries, it is him rather than anyone else that the doctor should listen to.

They [the doctors] listen to you more then, when there’s not a parent there trying to tell them [the doctors] what they have … what they feel … Yeah, it feels like it’s just me that they are listening to … Because, they are my worries, not anyone else’s, so it’s me that they should listen to, not to what someone else has noticed about it. (Carl, 19)

## Discussion

To the best of our knowledge, this is the first study that explores specifically adolescent males’ experiences of GP consultations without restrictions to certain kinds of visits or health concerns. We developed an overarching theme, *To be listened to*, and three themes: *To handle insecurity and uneasiness, To be understood and cared for*, and *To get parental support on his terms*. The adolescent males want GPs to take them seriously, to sincerely try to understand their worries, to care for them with interest and engagement, and to adapt appointment and advice to their needs and everyday life. They fear becoming embarrassed and being dismissed without getting any help, as they would then have exposed themselves as vulnerable in vain. They may need a parent as support, but their own view of the problem must have priority, and their privacy must be respected. The emphasis on being taken seriously in the overarching theme *To be listened to* implies an important, but not self-evident, feature consistent with other studies [7, 10, 15, 18] describing that adolescents are not taken seriously due to their age [7, 8, 41]. They want neither to be treated as children incapable of understanding what happens [7], nor as attention seeking teenagers going through a phase [7, 10], but as adults [15], or as Daley puts is: as a normal person [15]. Thus, *To be listened to* means that despite their young age, adolescent males want to be taken as seriously as anyone else.

The theme *To handle insecurity and uneasiness* reflects a vulnerability derived from his age, inexperience of GP consultations, and an ongoing neurocognitive development. Although logical reasoning is generally considered mature around age 16, the adolescent brain has an increased sensitivity to stress, embarrassment, and other emotions until the mid-20s, when the neurocognitive maturation is considered complete [42, 43]. In accordance with the literature, the adolescent males valued having enough time to understand what was taking place and what was being said, and to be able to ask when they did not [44, 45]. The present study, however, attributes the need of a slower pace to not yet acquired communication skills in a way not previously shown. Such communication difficulties, derived from the combination of age-related heightened susceptibility to emotions and a dearth of experience to draw upon, highlight the importance of allotting sufficient time in GP consultations. Both adolescents and GPs have reported time constraints as barriers to good adolescent healthcare in primary care [7, 10, 12]. The present study brings new light to the ubiquitous requests for more time by connecting them to age-appropriate inabilities.

The theme *To handle insecurity and uneasiness* also reflects a vulnerability related to notions of masculinity, suggesting that adolescent males may be more vulnerable and embarrassed in GP consultations than their female peers. Illness and help-seeking can be perceived as weaknesses contradictory to the notion of men being strong, independent, capable of dealing with pain, and able to solve the problem themselves [27, 28, 46, 47]. In the present study, such notions of masculinity were apparent in the descriptions of being taught not to reveal their pain or talk about feelings, and how they therefore struggled with being help-seeking and in pain while remaining a man.

Central to the theme *To be understood and cared for* is that the GP cares about him, understands him and adapts the appointment to his needs. It is well supported in the literature that adolescents prefer GPs with a caring, interested, and engaged approach, who make an effort for his or her benefit [7, 10, 11, 15, 17], and adapt their behaviour to the adolescent’s needs [10, 45]. What is new, however, is the description of adaption as a necessary condition of being understood and cared for. In other words, he determines whether the doctor cares about him and understands him by judging how well the doctor adapts the appointment to him and his needs.

Parental involvement in GP consultations are frequently mentioned in consultation studies, often in terms of confidentiality concerns [7, 11, 15, 44], but the present study brings forth the complexity of their role. During the consultation, the adolescent males balance between need of parental support, privacy and being the person the doctor listens to. They could also be rather indifferent to parental presence, describing that they would ask whatever they needed anyway, or lie if the truth was too embarrassing, presuming that their parents would not interfere until necessary. We were surprised to find that they appreciated the presence of and support from their parents to such a great extent, because the participating adolescent males were all in mid- or late adolescence, during which the value of parental support is known to decrease as independence grows [43]. They might have brought a parent to counter their fears of being misunderstood [7] or not being taken seriously [10]. Another possible explanation can be that the participating adolescent males mostly consulted for non-sensitive, physical complaints. Alternatively, if we assume a definition of autonomy as independence with maintained parental connectedness [48], the acceptance of parental involvement could be seen as a sign of connectedness instead of parental dependence and immature autonomy. The interpretation of the participants as independent while connected to their parents is strengthened by how the parents are assumed to stay in the background until needed, and by the adolescent male’s assertion that his view of the problem should have priority.

### Strengths and limitations

A strength of the study is the use of the lifeworld perspective, which allowed us to seek meaning in adolescent males’ own descriptions of their lived experiences of GP consultations without other theoretical assumptions. The goal was to see the phenomenon in all its variation, thus revealing valid, transferable meanings that are relevant and useful in other contexts [31, 40]. To ensure validity and rigour, openness, reflexivity, and a bridled preunderstanding were strived for [31, 40]. Throughout the research process, the authors sought to be sensitive to new and unexpected aspects and regularly discussed and critically questioned their emerging understanding [31, 38, 40]. To ensure quality, we followed Braun’s and Clarke’s guidelines for good reflexive thematic analysis [36, 39, 49].

Validity also requires education and experience [31], wherefore another strength of the study is the authors’ knowledge of context, field of research, and methodology. JH is a female PhD student and a GP employed at healthcare centre A. She is trained in research interviewing, and teaches consultation skills to medical students. She did not know any of the invited adolescent males, and introduced herself as a researcher. LN is a female qualitative researcher, well experienced in lifeworld theory and primary care research. LJ is a male GP, bioethicist, and qualitative researcher. YT is a female paediatrician teaching and researching adolescent medicine, and PK is a male GP, who researches and teaches consultation skills.

A third strength is the varied nature of the studied GP consultations, which widens the findings’ transferability. By exploring the general meanings of rather diverse experiences, we can understand central aspects of how GP consultations can be experienced by adolescent males, and transfer the findings to other adolescent males visiting GPs. Depending on the circumstances, the findings may also be transferable to adolescent males in other healthcare contexts.

A possible limitation is that a female, middle-aged GP interviewed the informants in a consultation room in the same healthcare centre where they had had their appointment. The setting might have aggravated the interview’s power imbalance and thus constrained the adolescent males’ narratives. This might explain why the adolescent males were surprisingly satisfied with the consultation [7, 8, 10, 15, 18]. However, the interviewer perceived their descriptions as honest and consistent. It is also possible [7, 10, 18] that the participating GPs had a higher-than-average interest in adolescents, or that the presence of a video recorder encouraged them to perform well. It could also be that the informants were not given enough time to reflect on their experiences before the interview; on the other hand, the interview that was conducted two weeks later did not differ particularly from the others. Furthermore, no consultations addressed mental or sexual health, a fact that could have reduced the demand on the GP’s communicative skills. Nevertheless, the informants did have to reveal embarrassing facts or endure painful examinations. A last reason for the unpredicted absence of negative descriptions might be the present study’s focus on specific consultations rather than allowing informants to describe consultations of their choice [7, 8, 18, 27]. It has been suggested that negative experiences are more vividly remembered [10, 18]. If such a causality exists, one would expect it to influence informants’ descriptions in more open-ended studies.

Another limitation is the rather small study sample. All informants contributed, however, to each theme, and sufficient information power was attained [35]. The descriptions might be positively skewed, but this does not detract from the adolescent males’ descriptions of what they value still being valid and transferable to similar contexts.

### Practical implications

Due to their ongoing neurocognitive and emotional development, inexperience, and notions of masculinity, we argue that adolescent males are particularly vulnerable. To take them as seriously as other age groups, GPs need to consider their inexperience and age-related heightened susceptibility to emotions, whilst still treating them as respectfully and seriously as they would adults. The adolescent male needs time to understand and interact with the GP, who must adapt language, pace, follow-up questions, examinations, explanations, and advice to him, so that he realizes that he has been understood and that the doctor cares about him. Sufficient time must therefore be allocated even for relatively simple medical issues.

Our findings also indicate that the embarrassment that adolescent males experience when revealing their troubles is compounded by traditional notions of masculinity [27, 47]. GPs can ease such embarrassment by using verbal or non-verbal emotional validation [50]. It has however been suggested that adolescent males interpret facial expressions differently than adults, more often finding neutral faces angry than adults do [51]. As non-verbal validation might be misunderstood, we recommend a generous use of verbal emotional validation, i.e. affirmative expressions conveying understanding such as “That must be hard for you” [50].

GPs may also need to be attentive to and assist the adolescent males’ complex balancing between independence, privacy, and parental support. Parental presence should not hinder the GP from listening primarily to the adolescent male. The GP may also offer private time without parents and explain what confidentiality means. How other consultation techniques affect adolescent males’ GP consultations, or how they best should be combined to meet the needs of adolescent males, were not within the scope of the present study, but would be an interesting area for future studies.

## Conclusions

Our findings illuminate that, in contrast to what is found in much of the literature, adolescent males can experience good GP consultations by rather simple means. The GP needs to care about him, to listen attentively, and take him seriously. Moreover, the GP must demonstrate his or her understanding throughout the appointment by adapting the consultation to his needs. It is through this adaptation to him as an individual that he realizes that he has been understood and cared for.

### Supplementary Information

Supplementary Material 1.

## Data Availability

To protect the study participants’ privacy, the full datasets used and analysed are not publicly available, but are available from the corresponding author upon reasonable request.

## Abbreviations

GP: General practitioner
