# Supplementary material for: They are my worries, so it’s me the doctor should listen to—adolescent males’ experiences of consultations with general practitioners
Source: BMC Prim Care. 2024 May 17;25:169. doi: 10.1186/s12875-024-02431-3 (PMC11102250; doi:10.1186/s12875-024-02431-3)
Supplement: Supplementary file 1 — Supplementary Material 1. [file 12875_2024_2431_MOESM1_ESM.docx]

# Interview guide

Translated to English by the authors.

For all seven areas of interest below, additional spontaneous questions were used to direct the attention of the adolescent male towards his lived experience of the GP consultation and to encourage him to speak openly. Moreover, appropriate follow-up questions were used to clarify and more deeply explore his experience such as: Can you tell me a bit more? Why is that important for you? How do you mean? What happened then? What did you feel? What did you think? Can you give me an example, please?

***1. Introduction.*** You have just met doctor (*name of the doctor*). How was it?

***2. A general description of his experience of the consultation.*** Can you tell me about the consultation, please? What happened?

***3. His experience of the doctor:*** Tell me about the doctor, please. How was he or she? Have you been to a doctor before? How was this one compared with the doctor(s) you have met before?

***4. His experience of the communication:*** How was the conversation? How did you feel talking to the doctor? Do you think that the doctor understood your troubles? Tell me why you think so, please. Did you feel understood? Did you understand what the doctor said? Did you have any opportunities to ask questions if needed? Did you get any help for your troubles?

***5. Experience of communication when parents or other relatives are present:*** I saw that your X (*mum, dad, girlfriend, etc*.) was present. How was it? In what ways do you think their presence affected the consultation?

***6. Negative experiences and suggestions for the future***. Is there anything that you wish was done differently? Was there anything that could have been better?

***7. Closing the interview.*** Is there anything that you would like to add? Is there anything that a researcher like me needs to know about how it is for a guy in your age to see a GP?
